# Nanocellulose/Fullerene Hybrid Films Assembled at the Air/Water Interface as Promising Functional Materials for Photo-electrocatalysis

**DOI:** 10.3390/polym13020243

**Published:** 2021-01-12

**Authors:** Francesco Milano, Maria Rachele Guascito, Paola Semeraro, Shadi Sawalha, Tatiana Da Ros, Alessandra Operamolla, Livia Giotta, Maurizio Prato, Ludovico Valli

**Affiliations:** 1 Istituto di Scienze delle Produzioni Alimentari (ISPA), Consiglio Nazionale delle Ricerche (CNR), S.P. Lecce-Monteroni, Ecotekne, 73100 Lecce, Italy; francesco.milano@cnr.it; 2 Dipartimento di Scienze e Tecnologie Biologiche e Ambientali, Università del Salento, S.P. Lecce-Monteroni, 73100 Lecce, Italy; maria.rachele.guascito@unisalento.it (M.R.G.); paola.semeraro@unisalento.it (P.S.); ludovico.valli@unisalento.it (L.V.); 3 Consorzio Interuniversitario Nazionale per la Scienza e Tecnologia dei Materiali (INSTM), Unità di Lecce, S.P. Lecce-Monteroni, 73100 Lecce, Italy; 4 Dipartimento di Ingegneria dell’Innovazione, Università del Salento, S.P. Lecce-Monteroni, 73100 Lecce, Italy; shadi.sawalha@unisalento.it; 5 Department of Chemical Engineering, An-Najah National University, P.O. Box 7, Nablus 00970, Palestine; 6 Center of Excellence for Nanostructured Materials (CENMAT) and INSTM, Unit of Trieste, Dipartimento di Scienze Chimiche e Farmaceutiche, Università di Trieste, via Giorgieri 1, 34127 Trieste, Italy; daros@units.it (T.D.R.); prato@units.it (M.P.); 7 Dipartimento di Chimica e Chimica Industriale, Università di Pisa, Via Giuseppe Moruzzi 13, 56124 Pisa, Italy; 8 Center for Cooperative Research in Biomaterials (CIC biomaGUNE), Basque Research and Technology Alliance (BRTA), Paseo de Miramón 182, 20014 Donostia San Sebastián, Spain; 9 Basque Foundation for Science, Ikerbasque, 48013 Bilbao, Spain

**Keywords:** cellulose nanocrystals, fullerene, fulleropyrrolidine, Langmuir-Schäfer deposition, voltammetry, photocurrent, photocatalysis

## Abstract

Cellulose nanomaterials have been widely investigated in the last decade, unveiling attractive properties for emerging applications. The ability of sulfated cellulose nanocrystals (CNCs) to guide the supramolecular organization of amphiphilic fullerene derivatives at the air/water interface has been recently highlighted. Here, we further investigated the assembly of Langmuir hybrid films that are based on the electrostatic interaction between cationic fulleropyrrolidines deposited at the air/water interface and anionic CNCs dispersed in the subphase, assessing the influence of additional negatively charged species that are dissolved in the water phase. By means of isotherm acquisition and spectroscopic measurements, we demonstrated that a tetra-sulfonated porphyrin, which was introduced in the subphase as anionic competitor, strongly inhibited the binding of CNCs to the floating fullerene layer. Nevertheless, despite the strong inhibition by anionic molecules, the mutual interaction between fulleropyrrolidines at the interface and the CNCs led to the assembly of robust hybrid films, which could be efficiently transferred onto solid substrates. Interestingly, ITO-electrodes that were modified with five-layer hybrid films exhibited enhanced electrical capacitance and produced anodic photocurrents at 0.4 V vs Ag/AgCl, whose intensity (230 nA/cm^2^) proved to be four times higher than the one that was observed with the sole fullerene derivative (60 nA/cm^2^).

## 1. Introduction

The Langmuir–Schäfer (LS) technique represents a powerful tool for achieving the deposition of organized thin films of organic materials. It entails the assembling of amphiphilic molecules in floating layers, usually at the air–water interface, and their transfer onto suitable solid substrates by horizontal lifting. The LS approach yields excellently organized architectures [1,2,3], with fine control over molecular orientation, providing monomolecular layers with the hydrophobic side of molecules facing the substrate and their hydrophilic moieties that are exposed to the external environment [4]. This characteristic makes the LS technique unique for the control over the film anisotropy [5,6].

The LS technique has been successfully extended to carbon nanomaterials, like carbon nanotubes [7,8] or fullerenes [9,10], aiming to improve their solid-state organization. The thin film processing of these materials was found to be rather difficult without a suitable co-dispersing agent, due to the strong tendency to form aggregates [11,12]. LS co-deposition of carbon nanotubes with conjugated polymers was a strategy that was used to disrupt nanotube aggregates and enhance their performances as functional materials for photoelectrodes [7,13]. In this approach, the π-stacking interaction between the conjugated polymer and carbon nanotubes was the promoting mechanism for the formation of the hybrid films.

Aiming at preparing stable colloidal suspensions of carbon nanomaterials in aqueous media, a sustainable method was recently proposed, which was based on the use of sulfated nanocellulose (nanocrystals or nanofibres) as dispersing agent for single- and multi-walled carbon nanotubes and reduced graphene oxide. This approach enabled the preparation of a conductive cellulose nanopaper [14]. The literature also reports blends of nanocellulose with other electroactive materials, including either conductive polymers, such as polypyrrole [15], PEDOT [16], and polyaniline [17], or carbon nanostructures, such as carbon nanotubes [18,19], reduced graphene oxide and nanodots [20,21,22,23], with the general scope of preparing nanostructured electrodes with enhanced mechanical performances and sustainability [24,25].

Discovered in the 1950s [26,27], the nano-sized forms of cellulose have recently gained growing interest for their outstanding chemical and mechanical properties [28,29,30,31,32]. Various approaches for isolating nanocellulose have been described [33], some of which are very attractive for their low environmental impact [34]. In this context, nanocellulose-based materials show great potential for industry [35], as the principles of green and blue economies demand new sources of sustainable materials in substitution of oil-derivatives, for high volume applications in sectors, such as water treatment [36], paint and coating industry, building, hygiene, and paper industry [37]. Besides, new intriguing and emerging applications concern the use of nanocellulose in hydrogels and aerogels [38,39], emulsion stabilizers [40], biocatalyst immobilizers [41], biosensors [42], drug delivery systems [43], adsorbents for contaminants [44,45], nanocomposites for environmental remediation [46], photonic films and transparent substrates for optoelectronic devices, as well as new nanostructured electroactive materials [47,48,49]. Indeed, nanocellulose can expose a large active area, is transparent, and it is harmless for human health and the environment [50,51].

The Langmuir technology, which has been suitably adapted to direct the assembly of water-dispersed species, has been used to achieve the two-dimensional organization of sulfated cellulose nanocrystals (CNCs), useful for the preparation of transparent or anti-reflection coatings [52,53,54]. This method exploited the electrostatic interaction of anionic CNCs dispersed in the subphase with cationic surfactants that were deposited at the air/water interface. The first and, so far, the only attempt to use this promising approach to prepare photo-electroactive nanocellulose-based LS films has been presented in our recent work [55], where we described the two-dimensional (2D) organization of CNCs that are driven by a cationic photo-active counterpart that was deposited at the air/water interface. Specifically, the ability of sulfated CNCs to produce multilayered architectures with cationic fulleropyrrolidines by a Langmuir–Schäfer approach has been assessed and demonstrated, leading to thin films that exhibit outstanding (photo)electrocatalytic properties [55]. CNCs are crystalline fibers featuring a high aspect ratio, a diameter that ranges from 5 to 50 nm, and a length between 100 and 500 nm, according to the standards of the Technical Association of Pulp and Paper Industry (TAPPI). The interaction of CNCs with the cationic fulleropyrrolidine (FP) film produced 2D-organization of the nanocrystals and it was associated with the enhancement of FP-mediated cathodic currents, both in dark and light conditions. This finding was taken as a proof of the CNCs impact on the supramolecular organization of FP, which resulted in improved functional properties. Before our contribution, C_60_-fullerene has appeared in combination with CNCs only as covalently immobilized moiety on the nanocellulose surface, producing cellulose-C_60_ dyads that are potentially useful for the photodynamic therapy [56,57]. However, the optoelectronic potential of this kind of hybrid material had been never evaluated. 

**Figure 1:**
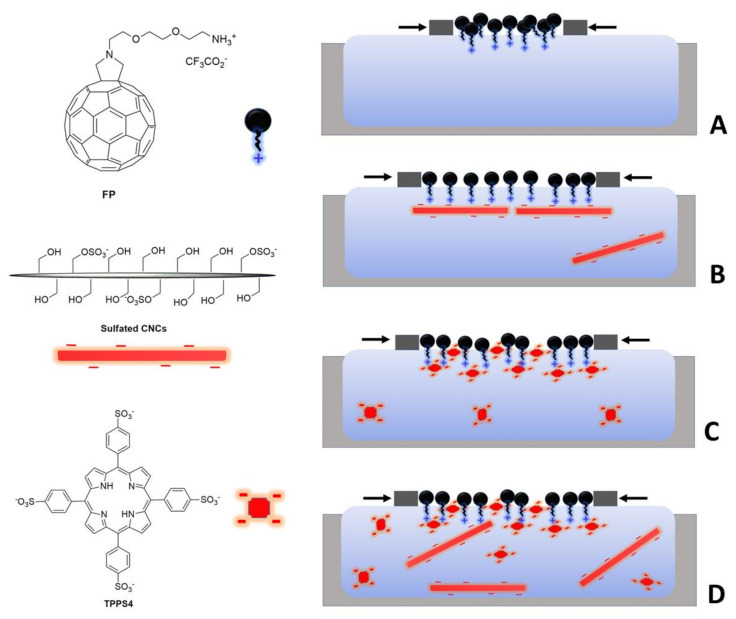
Left side: structure of the chemical entities employed in the present work, from the top to bottom: cationic fulleropyrrolidine (FP), sulfated cellulose nanocrystals (CNCs), meso-tetraphenylporphyrine-4,4′,4″,4″′-tetrasulfonate (TPPS4); only primary alcohol groups linked to C6 carbon of each anhydroglucose unit exposed to the surface of the nanocrystal are included in the representation. Right side: schematic representation of the FP floating layer compressed in the Langmuir trough to the same target surface pressure, using as subphases: pure water (**A**), CNCs 36 mg/L (**B**), TPPS4 3.6 mg/L (**C**) and a CNCs/TPPS4 blend (CNCs 36 mg/L + TPPS4 3.6 mg/L) (**D**). The closer distance between barriers in sketch A is indicative of a lower limiting area per FP molecule. The elements in the picture are not to scale.

Within the above-described scenario, the extensive characterization of the binding process at the air/water interface between sulfated CNCs and single-charged cationic FPs represents an interesting goal, when considering its impact on the functional properties of assembled materials. Therefore, this paper was addressed to this issue, by employing an original experimental design based on competitive processes investigation (Figure 1). Specifically, the electrostatic interaction between nanocellulose and FP Langmuir layers was probed by introducing in the subphase a CNCs competitor: the meso-tetraphenylporphyrine-4,4′,4″,4″′-tetrasulfonate (TPPS4). This anionic porphyrin was chosen due to its well-established ability to bind to amphiphilic cationic FPs that were layered at the air–water interface, enabling the deposition of porphyrin-FP dyads by an analogous LS approach [5,58,59]. Moreover, the sulfonate moieties that are present in TPPS4 structure are chemically similar to the sulfate groups of the proposed anionic CNCs. These competition experiments enabled us to establish the electrostatic nature of the FP/CNCs interaction at the air/water interface, as well as to highlight the role that is played by colloidal dimensions, aspect ratio, and charge density in directing the kinetic and thermodynamic control of the binding process. Additionally, we explored the electrochemical behavior of FP/CNCs hybrid films at anodic potentials, providing new and intriguing information on the strong ability of CNCs to boost the photo-response of fullerene-modified electrode surfaces.

## 2. Materials and Methods 

Figure 1 shows the structure of the cationic fullerene derivative employed in the present work and it was prepared as a trifluoroacetate salt, according to previously reported synthetic procedures [60]. The meso-tetraphenylporphyrine-4,4′,4″,4″′-tetrasulfonic acid (TPPS4 free acid) was purchased from Sigma-Aldrich (Saint Louis, MO, USA). The nanocrystalline cellulose was prepared while using Avicel PH-101 as a starting material following the procedure that was described in [61], while using the well-established sulfuric acid method [62]. Briefly, 5 g of Avicel PH-101 (Sigma-Aldrich, MO, USA) were warmed at 45 °C for 80 min in 80 mL 50 % *v/v* H_2_SO_4_ under mechanical stirring. The resulting mixture was diluted with deionized water (200 mL) and then purified by centrifugation at 1300 rcf for 10 min. The resulting suspension presented a pH of 3.90, as judged with a pH-meter, and a concentration of ~12 mg/mL, measured by subjecting known solution volumes to freeze-drying. Subsequently, the resulting suspension was dialyzed (cut off 12,400 Da) against distilled water until neutrality. The suspension was removed from the dialysis tube, sonicated with the aid of a Branson 250 tip sonicator (Danbury, CT, USA) (power 40 W, duty cycle 60%, time 10 min) and then centrifuged at 1300 rcf for 10 min. AFM measurements allowed for assessing that the final suspension contained nanocellulose rods with length 280 ± 70 nm, width 10 ± 2 nm, and aspect ratio 28. Elemental analyses on a freeze-dried sample of nanocellulose, which were carried out in triplicate, revealed a sulfur weight content of 0.9 ± 0.3%, corresponding to a total degree of substitution (DS) of 0.068.

In order to prepare the spreading solution for Langmuir film assembly, a suitable amount of the fulleropyrrolidine salt was weighted and then dissolved in few drops of dimethylsulfoxide (Fluka, GC grade, Munich, Germany); afterwards, chloroform (Fluka, HPLC grade, Munich, Germany) was added to reach the final concentration of 0.145 mg/mL. The resulting colloidal suspension was spread onto a water subphase while using a 601BAM trough (NIMA technology Ltd., Coventry, UK) with a 450 cm^2^ surface area. Ultrapure water, which was produced by a Milli-Q system (Merck Millipore, Burlington, MA, USA) (resistivity > 18 MΩ cm), was employed as control subphase and for the preparation of nanocellulose and TPPS4 supplemented subphases. For this purpose, CNCs were dispersed in water at the concentration of 36 mg/L, while TPPS4 was dissolved at a concentration of 3.6 mg/L (10^−6^ M), already assessed as optimal concentration for binding experiments in a previous work [58]. A blend of the two compounds, each of them at the above-mentioned concentration, was employed as subphase for competition experiments (see Figure 1, right side). After waiting 15 min, to allow for solvent evaporation, the floating films were compressed at a constant barrier speed of 7 mm/min, while monitoring the surface pressure by a Wilhelmy balance. Brewster angle microscopy (BAM) images of the floating films were acquired while using an BAM 2plus system (NFT, Göttingen, Germany) with a lateral resolution of 2 µm.

For LS deposition, the barriers were stopped at 18 mN/m surface pressure and a suitable Teflon grid was gently placed onto the film-covered water surface, thus dividing the continuous floating layer in multiple rectangular-shaped portions. Subsequently, the substrate was lowered horizontally in order to contact the film in each well and lift upwards very slowly, allowing for the transfer of the Langmuir layer on its surface. A gentle nitrogen stream was employed for drying the wet transferred film. These steps were repeated multiple times using the same slide to obtain multilayered films. Glass slides coated with indium tin oxide (ITO) were employed as substrates for Fourier Transform Infrared (FTIR) characterization and electrochemical measurements, while quartz slides were employed for UV-vis spectroscopy. In order to favor the interaction with the air-exposed hydrophobic side of the floating films, prior to the deposition, the substrates were exposed overnight to vapors of 1,1,1,6,6,6-hexamethyldisilazane.

Mid-infrared spectra (400–4000 cm^−1^ wavenumber range) of thin films deposited onto ITO-coated glass slides were acquired with a Spectrum One FTIR spectrometer (PerkinElmer, Waltham, MA, USA) that was equipped with a DTGS detector. A multireflection accessory, suitably designed for thin film analysis (AmplifIR, SensIR technologies, Danbury, CT, USA), was employed. Typically, 32 interferograms at 4 cm^−1^ resolution were acquired and then averaged for each spectrum. The Attenuated Total Reflectance (ATR)FTIR spectra of bulk solid samples were acquired by means of a horizontal sampling ATR apparatus that was equipped with a three-bounce diamond microprism as internal reflection element (PerkinElmer, Waltham, MA, USA). In this case, 16 interferograms were acquired and averaged for each spectrum.

UV-visible spectra were acquired with a Cary 5000 UV-vis-NIR spectrophotometer (Agilent Technologies, Santa Clara, CA, USA).

Electrochemical measurements were carried out by means of a µStat400 portable electrochemical sensor interface workstation (DropSens, Oviedo, Spain). A three-electrode configuration was adopted, where the counter electrode was a platinum wire and the working electrode (WE) was an ITO-coated glass slide modified with LS films of FP and FP/CNCs. An Ag/AgCl electrode was employed as the reference. The electrolyte medium contained 90 mM potassium phosphate at pH 7.0. Linear sweep voltammetry (LSV) and chronoamperometry measurements were conducted without any redox mediator addition and in aerobic conditions. The electrochemical photo-response was assessed, performing the measurements under illumination while using a 150 W quartz tungsten halogen lamp that was placed 5 cm from the surface of the WE.

## 3. Results and Discussion

### 3.1. Characterization of Films at the Air/Water Interface

**Figure 2:**
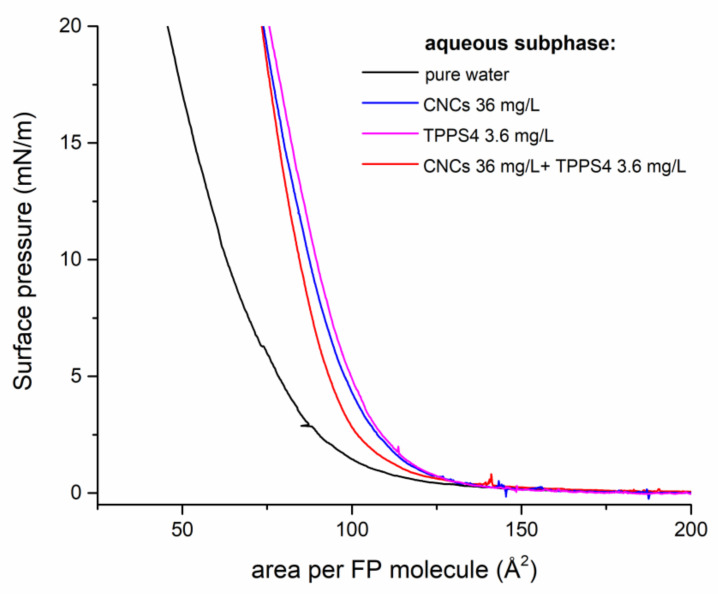
Langmuir isotherms of a floating FP film deposited on different aqueous subphases as indicated in the legend. Barrier speed during compression was 7 mm/min.

Figure 2 shows the Langmuir isotherms of FP, i.e., the graphs showing the surface pressure of the floating film as a function of the nominal area available for each FP molecule, recorded with different subphases. The isotherm shapes were consistent with a single compression-induced transition from a gas-like behavior of the spread material to a solid-like packed state. The limiting area value that was relevant to the solid state, which resulted from the intercept with the X-axis of the straight line interpolating the steep portion of the curves, increased when either CNCs or TPPS4, singularly or blended, were added to the water subphase. In detail, the same increment of 20 Å^2^ (from 85 to around 105 Å^2^) was observed when the pure water subphase was replaced by any of the three tested aqueous solutions, which proved to exert analogous effects on the surface activity of FP. The slight differences among the three isotherms that were relevant to supplemented subphases were indeed not significant, while taking the intrinsic repeatability of the measurement into account. 

**Figure 3:**
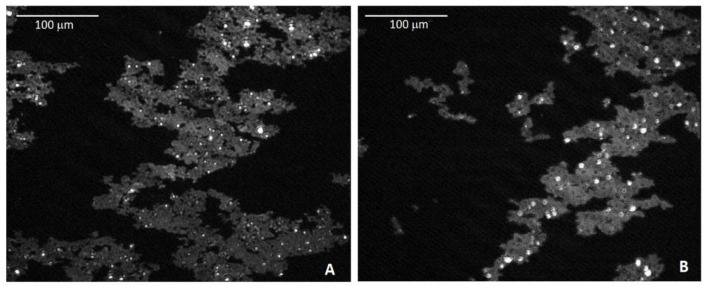
Brewster angle microscopy (BAM) images of a floating film of FP deposited onto pure water (**A**) and onto an aqueous subphase containing a blend of CNCs 36 mg/L and TPPS4 3.6 mg/L (**B**). Both micrographs are recorded at 0.5 mN/m surface pressure, although they are representative of the whole apparent gas-like phase (the aggregates appeared as soon as the spreading solvent evaporated). Bright spots on the top of planar aggregates represent colloidal insolubilized material. Floating films deposited on subphases singularly supplemented with CNCs and TPPS4 showed the same morphological features (data not shown).

FP cannot be considered to be an ideal amphiphilic substance and it does not behave as a two-dimensional gas at zero surface pressure, as already highlighted in previous reports [58]. Indeed, the strong π-π interactions among fullerene cages are responsible for large floating aggregates, which self-assemble as soon as the spreading solvent evaporates. Additionally, the presence of three-dimensional (3D) spherical aggregates, arising from insolubilized colloidal material and localizing on the top of the planar aggregates, was previously highlighted [55]. These features clearly appeared in the BAM images that are shown in Figure 3, which indicated that the morphology of our films at the microscopic scale was not affected by the specific subphase employed. Nevertheless, the rigidity of the solid film that results from the coalescence of FP aggregates was enhanced in the case of supplemented subphases, as demonstrated by the higher slope of the lines interpolating the steep portions of relevant isotherms. 

Therefore, the main information arising from the analysis of isotherms in Figure 2 is the analogous limit area increase that was observed while using either a molecular anionic system (TPPS4) or a colloidal anionic system (CNCs) and the absence of significant additive/synergic effects while using a subphase containing both systems, each of them at the same concentration employed singularly.

### 3.2. Characterization of LS Films 

The floating films relevant to isotherms of Figure 2 were transferred by horizontal lifting at the surface pressure of 18 mN/m onto ITO/glass substrates and then analyzed by mid-infrared spectroscopy. Despite the intrinsic low sensitivity of infrared spectroscopy, the high infrared reflectivity of ITO and the employment of a multireflection accessory allowed for acquiring good quality spectra, even in the presence of very thin layers. 

**Figure 4:**
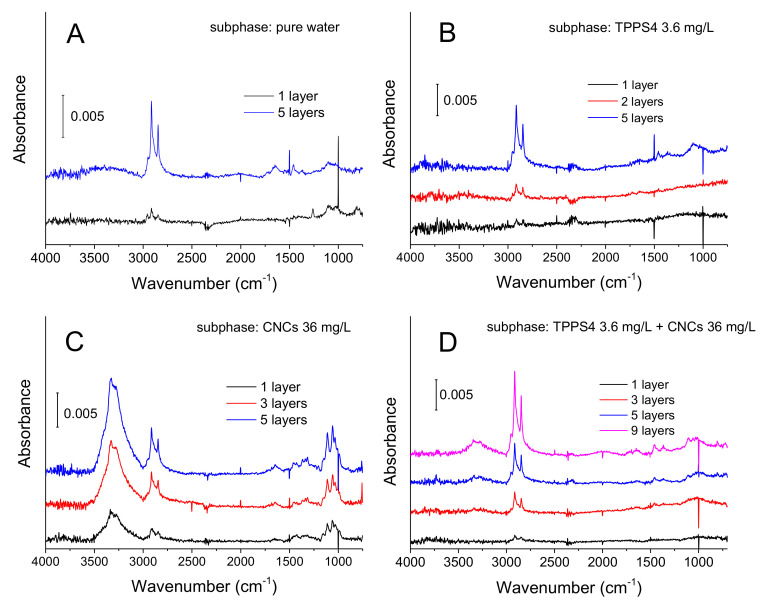
FTIR spectra recorded in transflectance mode of Langmuir-Schäfer FP films deposited at 18 mN/m surface pressure onto ITO/glass slides from the different subphases indicated in each panel: pure water (**A**); TPPS4 (**B**); CNCs (**C**); TPPS4 + CNCs (**D**). 32 interferograms were collected and averaged for each spectrum. The bare ITO substrate was employed for collecting the background spectrum.

Figure 4 shows the spectra of multiple LS layers of FP that were assembled on pure water and on subphases supplemented with CNCs and TPPS4, singularly or blended. As arises from panel A, the main spectral feature of transferred FP layers was represented by asymmetric and symmetric C–H stretching bands at 2915 and 2948 cm^−1^ ascribable to methylene groups that are present in the pyrrolidine functionality. Weaker absorption bands, which became better detectable at increasing number of layers, appeared at lower wavenumbers and could be ascribed to bending modes of >CH_2_ (~1460 cm^−1^) and to ether (~1100 cm^−1^) and amine (~1650 cm^−1^) group vibrations. Trifluoroacetate absorptions were not detectable, in agreement with significant FP deprotonation at the air/water interface because of acid-base equilibria perturbation [55,63].

**Figure 5:**
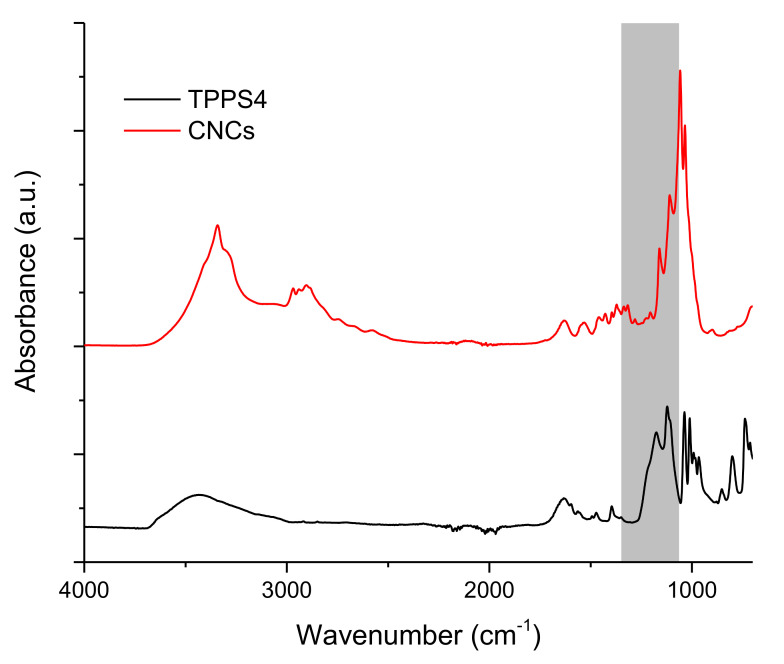
ATR-FTIR spectra of TPPS4 (black trace) and CNCs (red trace). The grey region represents the spectral window where sulfate/sulfonate groups absorb.

**Figure 6:**
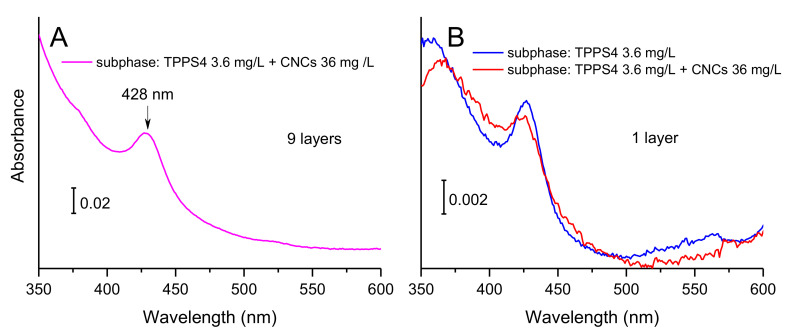
(**A**) Visible spectrum of a nine-layer Langmuir–Schäfer (LS) film of FP assembled on a blend of CNCs and TPPS4. (**B**) Visible spectra of single layers of FP transferred from a TPPS4 supplemented subphase (blue trace) and from a blend containing both TPPS4 and CNCs (red trace).

The spectral pattern of FP assembled on pure water subphase was substantially reproduced when a TPPS4-supplemented subphase was employed (Figure 4, panel B). Indeed, the typical –SO_3_^2−^ absorption bands of TPPS4 in the 1250–1100 cm^−1^ spectral window (see TPPS4 reference spectrum in Figure 5, black trace) were not detectable in FP/TPPS4 hybrid films, indicating that the amount of transferred porphyrin was very small. This finding pointed out the monomolecular characteristics of the TPPS4 layer formed by electrostatic interaction with the floating FP film. On the other side, the detectability of FP signals in plain and hybrid FP/TPPS4 LS films, even when a single layer was transferred, could be ascribed to the presence of three-dimensional aggregates in floating films, which was well highlighted in BAM images (Figure 3) and is responsible for a higher infrared absorption. However, the presence of the porphyrin in FP LS films that were assembled on a TPPS4 supplemented subphase was demonstrated by relevant visible spectra that were acquired in transmission mode, showing the typical Soret band of the tetrapyrrole ring at 428 nm (see blue trace in panel B of Figure 6).

Panel C in Figure 4 shows the mid-infrared spectra of hybrid films that were assembled on a CNCs supplemented subphase. The colloidal dimensions of CNCs co-transferred with FP by horizontal lifting accounted for the high intensity of nanocellulose absorption bands in these spectra, which were well detectable, even after a single transfer (black trace in panel C). The spectra were indeed dominated by the broad O–H stretching signal of cellulose at around 3300 cm^−1^ with further intense bands appearing at lower wavenumbers, between 1200 and 900 cm^−1^, due to acetal and alcoholic C–O bond vibrations. The spectrum of CNCs alone is shown for comparison in Figure 5 (red trace).

Interestingly, the typical infrared spectroscopic pattern of nanocellulose almost disappeared in films that were assembled on a subphase, where the additional species TPPS4 was present (see spectra in panel D of Figure 4). In fact, a small infrared absorption by CNCs could only be detected when multiple layers (more than 5) were transferred, as arisen from O–H and C–O bands that appear in blue and magenta traces in the same panel. Apart from this minor feature, the overall profile of infrared spectra of panel D reproduced the one of FP/TPPS4 hybrid films (panel B in Figure 4). Panel A in Figure 6 depicts the visible spectrum relevant to nine layers of FP assembled on the blend of CNCs and TPPS4. It clearly showed the typical Soret band of the aromatic macrocycle, confirming the presence of the porphyrin in the transferred film. Visible spectra that are relevant to one layer of FP assembled on a TPPS4 subphase, either alone or with the addition of CNCs (Figure 6, panel B), showed that the porphyrin bands were roughly overlapped, although a worsening of porphyrin transfer could be assessed in the presence of CNCs competitors.

**Figure 7:**
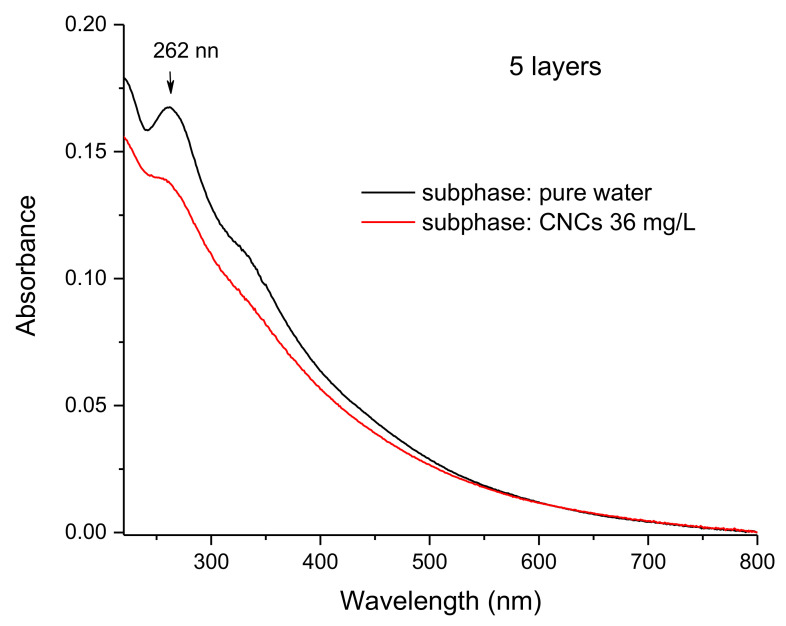
UV-visible spectra of LS films resulting from the horizontal transfer of five layers of FP assembled on a pure water subphase (black trace) and on a 36 mg/L colloidal suspension of CNCs (red trace).

In order to specifically investigate the ultraviolet region, where the major absorption of fullerene occurs, plain FP films and FP/CNCs hybrid films were deposited onto quartz slides and relevant UV spectra were collected (Figure 7). The spectra showed that CNCs induced a strong reduction of C_60_ absorption intensity in five-layer LS films, a phenomenon already observed in the mid-infrared range (when comparing panel A and C in Figure 4), but here much better highlighted due to the absence of any CNCs interfering bands. This finding further established that the assembly of FP onto CNCs highly enhanced the 2D character of floating films, which resulted in thinner and presumably more organized FP layers. 

### 3.3. Main Features of CNCs Binding Process Highlighted by Competition with TPPS4

We can safely conclude that the anionic porphyrin plays a key role in hampering the binding of CNCs to the FP floating layer based on the information that was gained from Langmuir isotherms and from visible and infrared spectra of relevant LS films. When employed as the subphase for FP Langmuir film assembly, the TPPS4/CNCs blend led indeed to LS films substantially equivalent to those that were obtained with a TPPS4 subphase. This effect may be rationalized when considering the main differences among the two anionic species, which affect both the thermodynamics and kinetics of the binding process.

From a thermodynamic point of view, assuming the interaction that was established at the interface of purely electrostatic nature, a greater Coulomb force is expected to act on TPPS4, due to its higher density of negative charge. In fact, the structure of TPPS4 presents four negatively charged sulfonate groups (Figure 1), which produced characteristic IR absorption bands in the 1370–1100 cm^−1^ wavenumber interval [64], with intensity that is comparable to the bands ascribable to the core of the structure (Figure 5, black trace. On the other hand, S=O stretching signals at 1033 cm^−1^ that arise from sulfate groups [65] were not clearly detectable in the ATR-FTIR spectrum of CNCs (Figure 5, red trace), which was instead dominated by the absorption bands of the sugar chains. This finding was consistent with a sulfation degree, calculated from nanocellulose elemental analysis, as low as 0.068 over an ideal value of 3 for fully sulfated hydroxyl groups on the cellulose backbone. The low sulfation degree indicated that the overall density of negative charge of the CNCs was significantly lower than the one that was exhibited by TPPS4, resulting in weaker Coulomb attraction by the cationic floating layer. It should be highlighted that π-π interactions among fullerene cages and the porphyrin aromatic macrocycle likely contributed to further strengthening the attractive interactions in the FP/TPPS4 hybrid film (Figure 1, sketch C).

In contrast, the electrostatic interaction guiding the two-dimensional organization of CNCs at the FP film/water interface could be mainly ascribed to the extended polyanionic nature of the nanocrystals, which favors the stabilization of the binding. However, unlike conventional polyelectrolytes, which present flexible chain structures, sulfated CNCs show the characteristic rigidity of crystalline materials. As one-dimensional (1D)-nanomaterials, they must organize beneath the floating FP layer with their long axis parallel to the interface in order to maximize attractive interactions that arise from multiple charges (Figure 1, sketch B). This peculiarity is expected to also play a crucial role in directing the organization of fullerene units spread at the air/water interface. 

In relation to kinetic aspects, it was evident that the colloidal dimensions and high aspect ratio of CNCs heavily affected the competition with TPPS4, accounting for a diffusion coefficient much lower than that of the porphyrin, a flat cross-shaped molecular species of relatively small size. The low diffusion coefficient of nanocrystals likely affected the migration rate of anionic nanocellulose from the bulk to the interface, under the Coulomb force field that is generated by FP ammonium groups. In the case of the binary system as subphase, the faster and tight binding of porphyrin units presumably modified the physico-chemical features of the floating layer with whom CNCs interacted. Specifically, the overall positive charge decreased, due to the neutralization of a certain amount of ammonium groups by the negatively charged sulfonate substituents of the porphyrin. Moreover, the attached macrocycles affected the geometrical profile of the hydrophilic side of the floating film, thus reducing its flatness. Consequently, the organization of nanocrystals parallel to the interface was hampered in the presence of bound porphyrin, due to both the attenuation of the electrostatic attraction (which could even switch to repulsion locally) and the steric hindrance owing to attached macrocycles (Figure 1, sketch D).

Taking the structural and mechanistic aspects discussed above into account, the observed strong inhibition of CNCs binding in the presence of TPPS4 could be strictly ascribed to the rigid nature of rod-shaped crystals, which did not allow for the adaptation of charged polymer chains to the specific surface characteristics, as in the case of conventional polyelectrolytes. On the other side, the adhesion of inflexible 2D-arranged crystals to the FP layer imparted stiffness to the floating film, as demonstrated by the higher slope of the solid-state branch of relevant isotherm, as compared to the one that was observed on a pure water subphase (Figure 2). The analogous increased rigidity of packed floating film in the presence of TPPS4 could be instead ascribed to porphyrin intercalation within FP films, as shown in Figure 1 (sketches C and D).

### 3.4. Photo-Electrochemical Characterization of FP/CNCs Hybrid Films 

**Figure 8:**
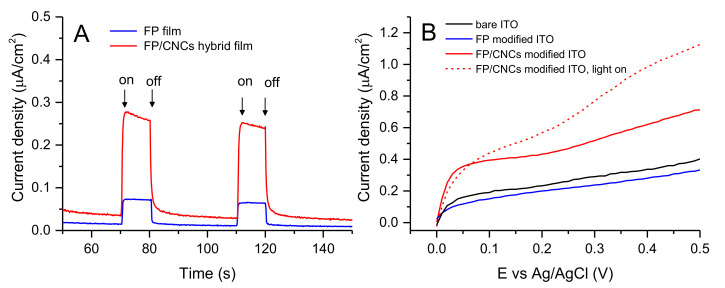
Chronoamperometry traces (**A**) recorded at 0.4 V vs Ag/AgCl applied potential using as working electrodes ITO (WEs ITO) electrodes modified with five layers of either FP assembled on pure water (blue line) or FP assembled on a CNCs 36 mg/L subphase (red line). Arrows indicate the times of light switching on and switching off. Linear sweep voltammetry (LSV) traces (**B**) recorded at 50 mV/s scan rate from 0.0 to 0.5 V relevant to the same FP and FP/CNCs films producing the chronoamperometries of panel A. The LSV trace of bare ITO (black line) has been added for comparison. The LSV recorded under light (dashed red line) has been shown only for FP/CNCs hybrid film since in the other cases the effect of light was negligible.

The rigidity of charged CNC rods likely contributed to ensuring the proper distance between bound fullerene cages, hampering the uncontrolled C_60_ aggregation and the consequent decline of its unique functional properties. In order to assess this desirable effect, the photo-electrochemical behavior of ITO electrodes coated with five layers of either FP or FP/CNCs was investigated in the neutral aqueous environment by chronoamperometry. The panel A shown in Figure 8 shows the time course of the current density across the cell at 0.4 V vs Ag/AgCl applied bias, alternating dark and light periods. The comparison between the two modified electrodes clearly highlighted the better performances of FP/CNCs hybrid film in the generation of anodic photocurrents (Table 1). A significant increase of the light-induced current increment was indeed observed, moving from the plain FP to the hybrid film, while only a slight change of the background current was detected.

In order to further investigate the electrochemical behavior of the films, linear voltammetries were recorded sweeping the potential from 0.0 to 0.5 V, both in dark and light conditions (panel B in Figure 8). Bare and FP-modified ITO electrodes presented a similar profile, which consists with comparable resistive and capacitive behavior of naked and FP-coated ITO surface. The observed profiles agreed with the typical electrochemical behavior of ITO in aqueous electrolyte, which shows a relatively featureless anodic region, with the earliest onset for the oxygen evolution reaction (OER) at 1.92 V vs RHE [66]. Moreover, consistently with our observation, Szucs et al. reported that electrochemical oxidation processes in C_60_ films do not occur in the investigated potential window, showing a flat voltammetry in the anodic side, unless the films were previously reduced at highly negative potentials [67], which was not our case. In contrast, the LSV trace that is relevant to FP/CNCs hybrid film (red line in panel B of Figure 8) exhibited a significant increase of the current flowing across the external circuit during the potential scan from 0.0 to 0.5 V vs Ag/AgCl. This behavior highlighted both the important enhancement of the electrical capacity of the coating layer and its ability to promote faradaic processes, whose occurrence at potentials beyond 0.25 V was suggested by the slight increase of the slope of the current/potential curve. Interestingly, the irradiation of the FP/CNCs hybrid film with visible light resulted in a noteworthy enhancement of current density at higher potentials, which indicated that CNCs, transparent to visible light, play an indirect but crucial role on the electrode photo-response. Instead, light irradiation produced negligible effects on bare ITO and FP/ITO electrodes (data not shown).

CNCs were found to increase the limiting area per FP molecule value (Figure 2) and, at the same, reduce the fulleropyrrolidine amount transferred on the solid substrate (Figure 7) for each LS run, as discussed above. These two events strongly supported the hypothesis that the nanocrystals favor the two-dimensional character of the film controlling mutual distances and interactions among C_60_ cages, thus ensuring a homogeneous C_60_ energy level landscape over the film surface. Moreover, by stabilizing FP ammonium terminal groups, anionic CNCs enhanced the ionic character of the film, improving its wettability, capacity, and electrical conductivity. In this scenario, a proper photo-activation of electron shuttling was expected, in nice agreement with the observed enhanced electrochemical photo-response. However, based on the results presented here, final conclusions about the specific faradaic process that is responsible for anodic current generation by FP/CNCs-modified electrodes could not be reached. The electrolyte medium composition and the chemical characteristics of the hybrid film suggested either water or nanocellulose itself as a possible electron-rich species able to sustain the anodic current. C_60_ HOMO and LUMO energy values (−6.30 and −4.46 eV, respectively [68]) indicate that excitation by visible light would, in principle, enable the mediation of the electron transfer from water to the ITO surface, thus favoring OER. The absolute standard potential energy that is relevant to the water oxidation half-reaction at pH 7 is indeed −5.32 eV. However, despite the well-known electron acceptor properties, which makes C_60_ the ideal counterpart of photoactive donor molecules in dyads performing photo-induced electron transfer [69,70], the possible application of C_60_ films for water oxidation photo(electro)catalysis has rarely been discussed in the literature [71,72]. A recent work reported on the employment of buckminsterfullerene adsorbed onto single-walled carbon nanotubes (SWCNT) as a multifunctional catalyst for oxygen reduction, oxygen evolution, and hydrogen evolution reactions [73]. Specifically, C_60_ alone was found to be rather inactive as a catalyst for electrochemical water splitting reactions, regardless its excitation by visible light. In contrast, outstanding catalytic activity was observed when C_60_ was adsorbed onto SWCNT. On the other side, nanocellulose oxidation would be thermodynamically even more favored than water oxidation, given the standard potential of glucose full oxidation half-reaction (−0.001 V vs RHE [74]). Moreover, the cellulose photo-reforming that is assisted by inorganic photocatalysts has been reported in the literature [75], suggesting a possible role of photo-excited FP in nanocellulose oxidation catalysis. In our previous work, we have shown that ITO electrodes that are modified with the same FP/CNCs hybrid film also act as photocathodes catalyzing the electrochemical hydrogen evolution reaction (HER). In this case, the role that was played by the ammonium terminal groups on the fulleropyrrolidine appendage was highlighted [55]. Therefore, whatever the faradaic process that is involved in the anodic side, the hybrid FP/CNCs material appeared to be very attractive in the field of photo-electrocatalysis. Indeed, it showed great potential as bifunctional (photo)catalyst for water splitting or biomass photo-reforming applications, both being very intriguing for their impact on renewable energy technology.

Finally, being highly desirable the optimization of catalytic performances under mild conditions, the ability of our hybrid film to express its catalytic properties at a neutral pH represents a further noteworthy advantage.

## 4. Conclusions

In this work, we have carried out a study on the competitive electrostatic interaction by sulfated CNCs and the porphyrin TPPS4 at the interface with the floating layer of a positively charged fulleropyrrolidine. The polyelectrolyte nature of CNCs, combined with their typical crystallinity, led to an electrostatically driven interaction with FP, which is able to exert a strong action on the 2D-organization of the FP Langmuir layer itself. However, the kinetics of the overall binding process of CNCs with FP proved to be slow and it suffered from competition with relatively small anionic molecules. Our data suggested that TPPS4 advantages from a higher mobility in water and reaches the FP layer sooner than the nanocrystals. Consequently, in the presence of TPPS4, a much smaller amount of nanocrystals binds to FP and can be lifted from the interface, producing LS films with negligible nanocellulose content.

Nevertheless, in the absence of competitor molecules, CNCs effectively bind to FP Langmuir layers, leading to robust and organized LS hybrid films, which proved to enhance the electrical photo-response of ITO electrodes at anodic potentials. The impact of CNCs addition on the photo-electrochemical activity of FP Langmuir films pointed out a new and appealing application of this renewable nanopolymer in the field of functional materials development. The results presented here encourage further investigations in order to elucidate current generation mechanisms and optimize long-term stability and intensity of the electrical output. The analysis of oxidation products that arise from anodic photo-current generation will shed light on the real potential of this all-organic 2D catalyst in the field of solar energy-driven electricity and biofuel production. In this context, the intrinsic renewability of nanocellulose crystals would make the hybrid films described here a promising alternative to inorganic photocatalysts.

## Figures and Tables

**Table 1:** Current densities recorded on ITO electrodes modified with FP and FP/CNCs films at 0.4 V vs Ag/AgCl applied potential.

LS Film	Background Current Density ^1^ (nA/cm^2^)	Current Density Under Illumination (nA/cm^2^)	Photocurrent (nA/cm^2^)
Five-layer FP	15	75	60
Five-layer FP/CNCs	30	260	230

^1^ After 30 s stabilization.

## Data Availability

The data presented in this study are available on request from the corresponding author.
